# What can go wrong when observations are not independently and identically distributed: A cautionary note on calculating correlations on combined data sets from different experiments or conditions

**DOI:** 10.3389/fsysb.2023.1042156

**Published:** 2023-01-30

**Authors:** Edoardo Saccenti

**Affiliations:** Laboratory of Systems and Synthetic Biology, Wageningen University and Research, Wageningen, Netherlands

**Keywords:** covariance, data fusion, data merging, pearson’s correlation, repeated measures, spearman’s correlation, technical replicates, violation of statistical assumptions

## Abstract

In the scientific literature data analysis results are often presented when samples from different experiments or different conditions, technical replicates or times series are merged to increase the sample size before calculating the correlation coefficient. This way of proceeding violates two basic assumptions underlying the use of the correlation coefficient: sampling from one population and independence of the observations (independence of errors). Since correlations are used to measure and infer associations between biological entities, this has tremendous implications on the reliability of scientific results, as the violation of these assumption leads to wrong and biased results. In this technical note, I review some basic properties of the Pearson’s correlation coefficient and illustrate some exemplary problems with simulated and experimental data, taking a didactic approach with the use of supporting graphical examples.

## 1 Introduction

The Pearson’s correlation coefficient (Pearson, 1895; Spearman, 1907) is certainly one of the most popular measures of association used in biology and in the Life Sciences. Unfortunately, it is also one of the most misused. Recently, several papers have been brought to my attention by collaborators in which the sample correlation coefficient is calculated following questionable practices, in particular when data from different experiments or conditions are combined before calculating the correlation. The goal of this cautionary note is to show what happens when the basic assumptions underlying the calculation and the use of the sample correlation are not met.

To set the scene, I start by introducing some notation and by recalling some basic statistical principles. Taken *n* observations (*x*_1_, *x*_2_, *…* , *x*_*n*_) of a variable *x* and *n* observations (*y*_1_, *y*_2_, *…* , *y*_*n*_) of a variable *y*, the Pearson’s sample correlation coefficient *r* ^(*xy*)^ (to which I will term “sample correlation” for sake of simplicity) is defined as

$$r^{\left(xy\right)}=\frac{C^{\left(xy\right)}}{\sqrt{V^{\left(x\right)}}\sqrt{V^{\left(y\right)}}},$$ (1)

where *V* ^(*x*)^ is the sample variance^1^

$$V^{\left(x\right)}=\frac{1}{n-1}\overset{n}{\underset{i=1}{\sum }}{\left(x_{i}-M_{n}^{\left(x\right)}\right)}^{2}\;\;V^{\left(y\right)}=\frac{1}{n-1}\overset{n}{\underset{i=1}{\sum }}{\left(y_{i}-M_{n}^{\left(y\right)}\right)}^{2},$$ (2)

and *C* ^(*xy*)^ is the sample covariance:

$$C^{\left(xy\right)}=\frac{1}{n-1}\overset{n}{\underset{i=1}{\sum }}\left(x_{i}-M_{n}^{\left(x\right)}\right)(y_{i}-M_{n}^{\left(y\right)})$$ (3)

with $M_{n}^{\left(x\right)}$ and $M_{n}^{\left(y\right)}$ the sample mean for *x* and *y*:

$$M_{n}^{\left(x\right)}=\frac{1}{n}\overset{n}{\underset{i=1}{\sum }}x_{i}\;\;M_{n}^{\left(y\right)}=\frac{1}{n}\overset{n}{\underset{i=1}{\sum }}y_{i}.$$ (4)

Let’s now recall now some facts about the sample correlation *r* ^(*xy*)^ (Eq. 1): it can always be calculated, however its validity and (correct) interpretation (including its statistical significance, as expressed by the associated *p*-value) rest on several statistical assumptions. I will focus here on the two assumptions stating that the observations used to calculate the correlation must be identically and independently distributed; these formulated as.

A1 All the (*x*_1_, *x*_2_, *…* , *x*_*n*_) observations of *x* (and *y*) are sampled from the same (normal) distribution.

A2 The (*x*_1_, *x*_2_, *…* , *x*_*n*_) observations of *x* (and *y*) are independent (independence of errors).

To be of any significance, and to be able to make inference on population parameters, the observations must be randomly selected, that is must be a representative random sample of a larger population. In addition, the relationships between *x* and *y* must be linear, since (Eq. 1) cannot account for non-linear relationships. Chapter 32 of Motulsky. (2014) offers a low level yet very precise presentation of all the assumptions underlying the use of the correlation coefficient.

This note deals with the problems arising when assumptions A1 and A2 are violated, that is when observations are non-independently and non-identically distributed. Consequences of the violations of other assumptions, like deviation from normality of the observations, have been discussed elsewhere (Calkins, 1974; Havlicek and Peterson, 1976; Havlicek and Peterson, 1977; Wilcox, 2009). The papers by Schober et al. (2018) and Janse et al. (2021) discuss pitfalls and interpretative problems of correlations.

In what follows, all observations are well-behaved and follow a normal (Gaussian) distribution:

$$x_{i}∼N\left(\mu ,\nu^{2}\right)$$ (5)

where *μ* and *ν* are the population mean and standard deviation (same for variable *y*).

## 2 Three research scenarios when correlations are wrongly calculated

I will consider three research scenarios often found in literature in which data is manipulated in some way before the correlation among measured variables is calculated.

RS1 A researcher has two data sets A and B which contain measurements of the variables *x* and *y*. Data set A contains *n* observations of *x* and *y*, while data set B contains *m* observations. A and B are combined into one data set containing *n* + *m* observations and the Pearson’s sample correlation (Eq. 1) is calculated between *x* and *y*. The reasons for merging the two data sets can be different. Data sets A and B can come from different batches measured during an experiment, or even from different experiments. Often the data sets are merged to increase the sample size with the idea (wrong, in this case) of obtaining a more a reliable estimation of the correlation between *x* and *y*.
RS2 A researcher has performed an experiment where a large number of variables have been measured over two conditions, on *n* observations for Condition A and *m* observation for Condition B, like, for example, in the case of a transcription experiment where thousands of gene expressions have been measured on case and control samples. To reduce the dimensionality of the problem, the researcher restricts the analysis to those genes that are differentially expressed between the two conditions. Moved by the interest of understanding regulatory mechanisms, the researcher decides to build a correlation network using all the measurements (observations) available for those genes (let’s call two of such genes *x* and *y*) that are differentially expressed.
RS3 A researcher has measured variable *x* and *y* several times on the same subjects, obtaining *m* measures for each subject. A typical case is when technical replicates are measured for each (or some of the) sample, usually in duo or triplicates, or when time series are acquired. To increase the total sample size, they then decide to combine all the *n* × *m* observations and calculate the correlation over the *n* × *m* observation of *x* and *y*.

**FIGURE 1:**
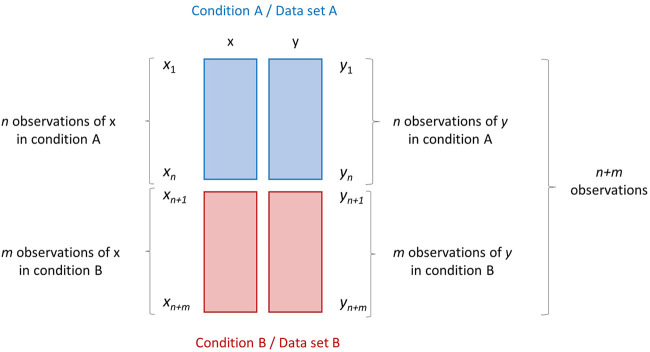
Research scenarios RS1 and RS2. Graphical illustration of two data sets A and B (depicted as blocks of different color) containing measurements/observations of two variables *x* and *y* measured on two different conditions. Data set A contains *n* observations, while data set B contains *m* observations. The data set obtained by merging A and B row-wise (on top of the other) has dimension (*n* + *m*) × 2.

The first two scenarios RS1 and RS2, albeit different, can be schematized in the same way, as shown in Figure 1.

### 2.1 Violation of sampling from one population

**FIGURE 2:**
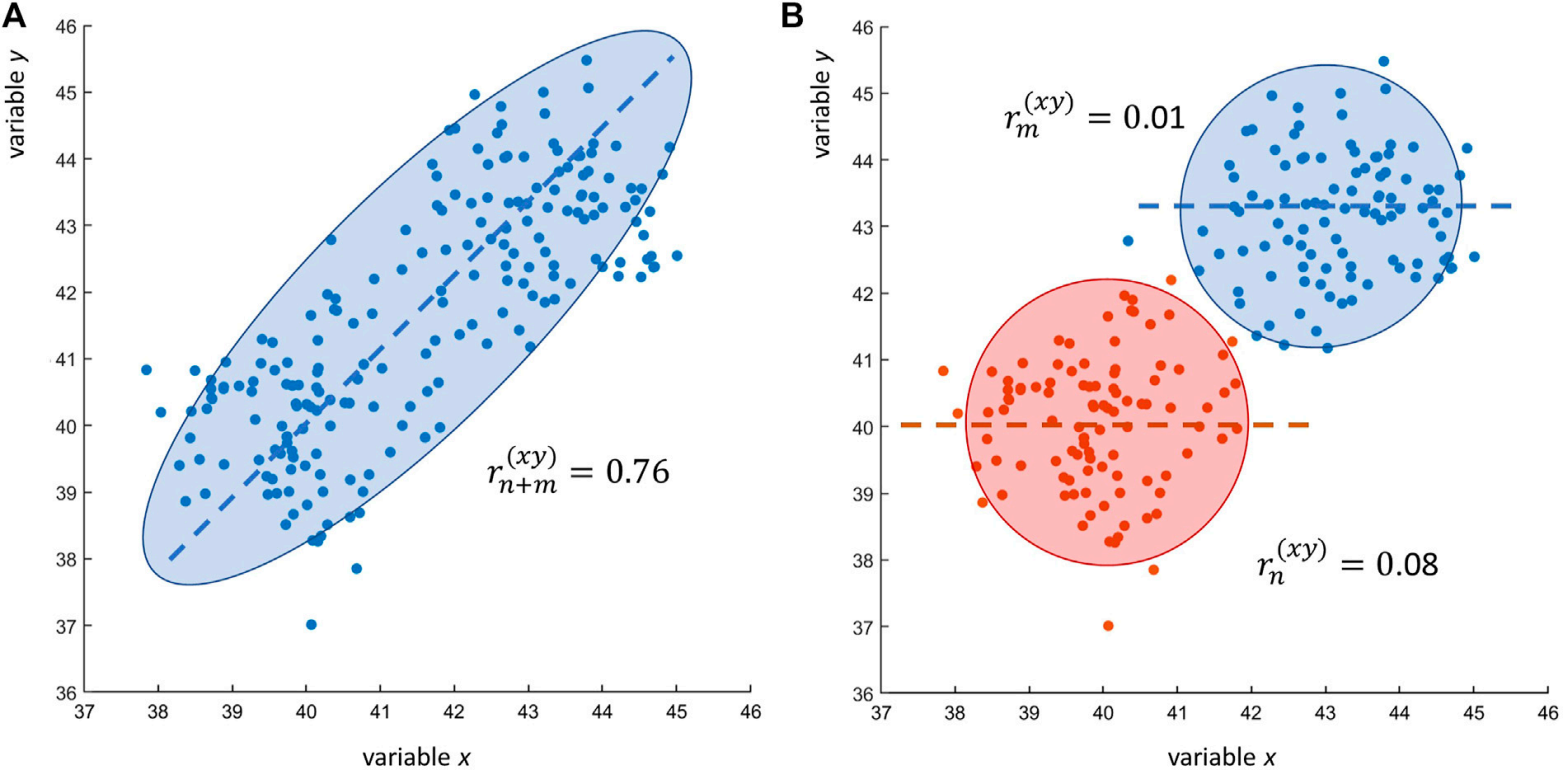
Research scenarios RS1 and RS2. **(A)** Scatter plot of *n* + *m* = 200 observations of two variables *x* and *y*: the Pearson’s sample correlation between *x* and *y* is $r_{n+m}^{\left(xy\right)}=0.76$ . **(B)** The same scatter plot as in Panel A but with data points color coded to highlight the actual data structure: when taken separately the *n* = *m* = 100 observations of *x* and *y* (Condition A: blue; condition B: red) are uncorrelated: $r_{n}^{\left(xy\right)}=0.01$ (data set A) and $r_{n}^{\left(xy\right)}=0.08$ (data set B). The observed high correlation $r_{n+m}^{\left(xy\right)}=0.76$ is an artifact due to the merging of two data set containing variables coming from two different populations: in this case *x* and *y* come from two independent normal distributions with population means *μ*_*A*_ = (43, 43) and *μ*_*B*_ = (40, 40) and unit variance *ν* ^2^ = 1.

Scenarios RS1 and RS2 entail the calculation of sample correlations after merging of two (or more) data sets: I wish to illustrate the problems that arise from such a way of operating with a simple simulated example. Figure 2A shows the correlation plot of *n* + *m* = 200 observations of variables *x* and *y*. A positive linear relationship seems to exist between *x* and *y*: the sample correlation coefficient is $r_{\left(n+m\right)}^{xy}=0.76$ (*P-val* $<10^{-5}$ ). Equipped with this rather strong correlation and statistical significance, the researcher may claim association between the two variables and build a story around it, explaining and discussing the biological relevance of it.

The problem becomes evident when we look at how the data presented in Figure 2B has been built. I have proceeded as described in research scenarios RS1 and RS2, merging two data sets containing *n* = 100 and *m* = 100 observation of *x* and *y* and then I have calculated the correlation between *x* and *y*. The reality is that *x* and *y* are not correlated at all: when the two data sets (conditions) are considered separately, the correlation between *x* and *y* is zero, since the generating mechanism of the data shown in Figure 2A is the following:

$$\begin{array}{l} \left(x_{1<i<n},y_{1<i<n}\right)∼N\left(40,1\right)\\ \left(x_{n+1<i<n+m},y_{n+1<i<n+m}\right)∼N\left(43,1\right), \end{array}$$ (6)

which generates variables *x* and *y* that are independent and uncorrelated. The second scenario RS2 is very often encountered in papers dealing with the analysis of very large omics data sets. This way of proceeding is also problematic. In fact, when the researcher looks for differentially expressed genes, (or for metabolites with different concentrations), they perform some statistical test to compare the observed means of variable *x* and *y* in condition A *versus* condition B (in this case a *t*-test, for instance), testing the Null hypothesis (similar considerations hold for variable *y*):

$$H_{0}:\mu_{A}^{\left(x\right)}=\mu_{B}^{\left(x\right)},$$ (7)

against the alternative

$$H_{1}:\mu_{A}^{\left(x\right)}\neq \mu_{B}^{\left(x\right)}.$$ (8)

There is of course no problem using the *t*-test to find genes that are differentially expressed (even if more powerful approaches have been introduced for this type of data). The problem arises when the differentially expressed genes are used to compute correlations. Selecting the variables for which H_0_ is rejected is the equivalent of selecting variables for which the distribution of *x* is different between two conditions. Stated in other words, by doing so the researcher is looking specifically for those variable that violates the assumption of sampling from one distribution!

**FIGURE 3:**
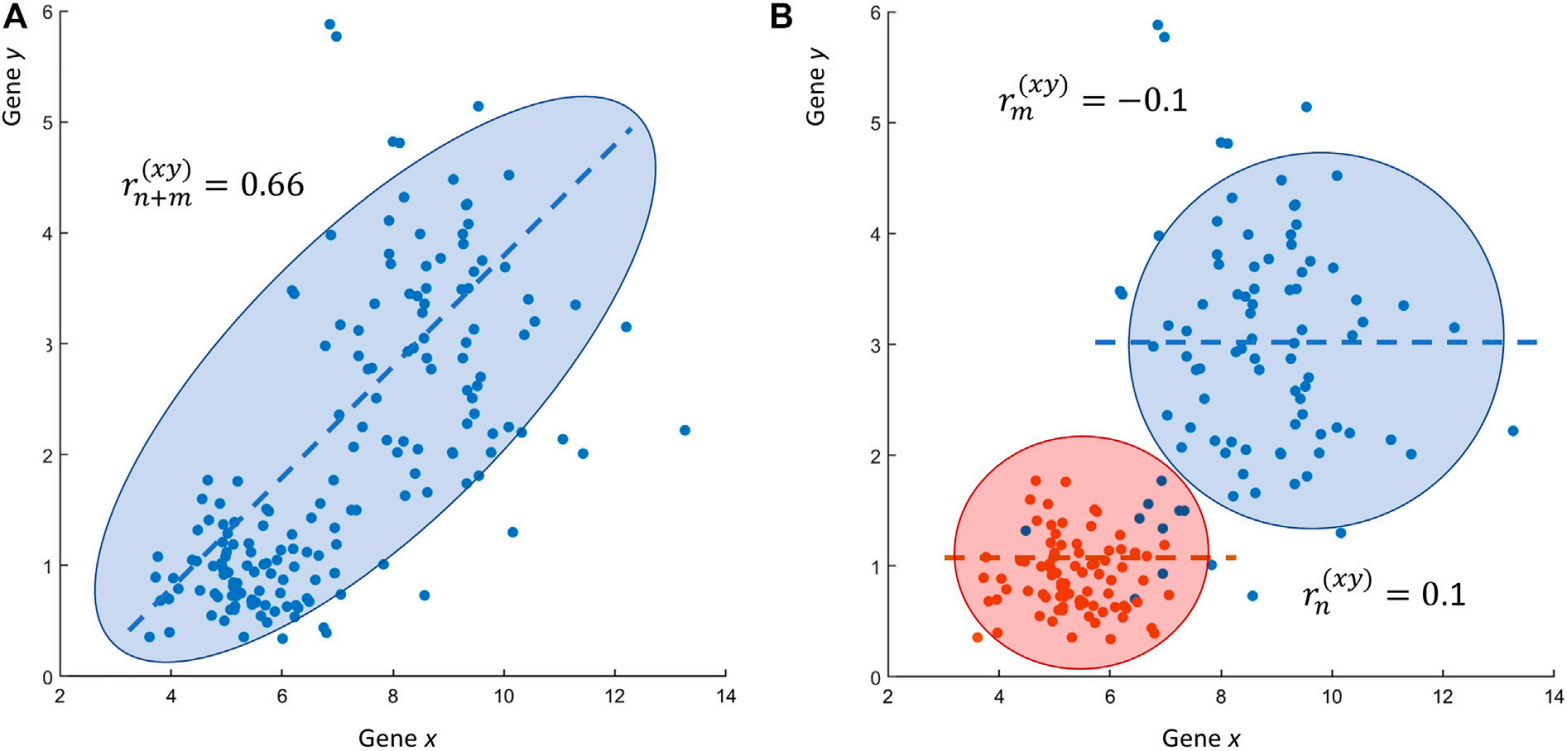
Real life research scenario RS2. **(A)** Scatter plot of the expression values of two genes *x* and *y*: the Pearson’s sample correlation between gene *x* and *y* is $r_{n+m}^{\left(xy\right)}=0.66$ . **(B)** The same scatter plot as in Panel A but with gene expression color coded to highlight the actual data structure: when taken separately the expression of genes *x* and *y* (Condition A: blue; condition B: red) are uncorrelated: $r_{m}^{\left(xy\right)}=-0.1$ and $r_{n}^{\left(xy\right)}=0.1$ , respectively. The observed correlation $r_{n+m}^{\left(xy\right)}=0.66$ is an artifact due to the merging of the expression profile of two genes that have been found to be differentially expressed between condition A and B (case and controls) (*P*-val $<10^{-5}$ ) violating, in this way, the assumption of sampling from the same distribution. Data from (Li et al., 2014). Note that there is nothing wrong with analysis of the data in the original publication: here the data has been only used to build an illustrative example.

If the reader thinks that these are just simulated numerical examples, it is not complicated to show that such problematic situations can be easily encountered when using real-life experimental data. Figure 3 shows a case similar to the one given in Figure 2, this time obtained using data from a transcriptomic study: the expression profiles of two genes *x* and *y*, measured at two different conditions in a case-control scenario, are uncorrelated ( $r_{n}^{\left(xy\right)}=0.1$ , $r_{m}^{\left(xy\right)}=-0.1$ ) when the two conditions are considered separately Figure 3A. However, they become correlated ( $r_{n+m}^{\left(xy\right)}$ , *P-val* $<10^{-5}$ ) (Figure 3B) if the correlation is taken over all the observations combined, i.e., when the two data sets are combined.

### 2.2 A closer look to the mathematics of the problem

This section presents a mathematical explanation of what observed in Figures 2, 3. Let’s define the overall mean $M_{m+n}^{\left(x\right)}$ over all the (*x*_1_, *x*_2_, … *x*_*n*+*m*_) observations of *x* (identical formulas hold for *y*)

$$M_{n+m}^{\left(x\right)}=\frac{1}{n+m}\overset{n+m}{\underset{i=1}{\sum }}x_{i},$$ (9)

and the partial means $M_{n}^{\left(x\right)}$ and $M_{m}^{\left(x\right)}$ over the (*x*_1_, *x*_2_, … *x*_*n*_) and (*x*_*n*+1_, *x*_*n*+2_, … *x*_*n*+*m*_), as

$$M_{n}^{\left(x\right)}=\frac{1}{n}\overset{n}{\underset{i=1}{\sum }}x_{i}$$ (10)

$$M_{m}^{\left(x\right)}=\frac{1}{m}\overset{n+m}{\underset{i=n+1}{\sum }}x_{i},$$ (11)

with similar definition for variable *y*. The overall variance $S_{n+m}^{\left(x\right)}$ of *x*_*n*+*m*_ is given by (Chan et al., 1982)

$$V_{n+m}^{\left(x\right)}=\frac{1}{n+m-1}\overset{n+m}{\underset{i=1}{\sum }}{\left(x_{i}-M_{m+n}^{\left(x\right)}\right)}^{2},$$ (12)

and the partial variances $S_{n}^{\left(x\right)}$ and $S_{m}^{\left(x\right)}$ by

$$\begin{array}{l} V_{n}^{\left(x\right)}=\frac{1}{n-1}\overset{n}{\underset{i=1}{\sum }}{\left(x_{i}-M_{n}^{\left(x\right)}\right)}^{2}\\ V_{m}^{\left(x\right)}=\frac{1}{m-1}\overset{n+m}{\underset{i=n+1}{\sum }}{\left(x_{i}-M_{m}^{\left(x\right)}\right)}^{2}. \end{array}$$ (13)

The total variance $S_{n+m}^{\left(x\right)}$ taken over all the observations of *x* can be expressed as function of the partial variances (Eq. 13) of the subsets

$$V_{n+m}^{\left(x\right)}=\frac{1}{n+m-1}\left[\left(n-1\right)V_{n}^{\left(x\right)}+\left(m-1\right)V_{m}^{\left(x\right)}+\frac{nm}{n+m}{\left(M_{n}^{\left(x\right)}-M_{m}^{\left(x\right)}\right)}^{2}\right].$$ (14)

The covariance $C_{m+n}^{\left(xy\right)}$ between the *n* + *m* observations of *x* and *y* can be expressed in term of the partial covariance $C_{n}^{\left(xy\right)}$ and $C_{m}^{\left(xy\right)}$ between the *n* (and *m*) observations of *x* and *y*:

$$C_{n+m}^{\left(xy\right)}=\frac{1}{n+m-1}[\left(n-1\right)C_{n}^{\left(xy\right)}+\left(m-1\right)C_{m}^{\left(xy\right)}+\frac{nm}{n+m}\left(M_{n}^{\left(x\right)}-M_{m}^{\left(x\right)}\right)\left(M_{n}^{\left(y\right)}-M_{m}^{\left(y\right)}\right)].$$ (15)

By combining (Eq. 15) with (Eqs 13, 14) it is possible to obtain the sample correlation $r_{n+m}^{\left(xy\right)}$ between *n* + *m* observations *x* and *y* as a function of the correlation $r_{n}^{\left(x\right)}$ and $r_{m}^{\left(x\right)}$ calculated on the two subsets (Hayes, 2012):

$$\begin{array}{ll} r_{n+m}^{\left(xy\right)} & =\frac{C_{n+m}^{\left(xy\right)}}{\sqrt{V_{n+m}^{\left(x\right)}}\sqrt{V_{n+m}^{\left(y\right)}}}\\ =\frac{1}{n+m-1}\frac{\left(n-1\right)C_{n}^{\left(xy\right)}+\left(m-1\right)C_{m}^{\left(xy\right)}+\frac{nm}{n+m}\left(M_{n}^{\left(x\right)}-M_{m}^{\left(x\right)}\right)\left(M_{n}^{\left(y\right)}-M_{m}^{\left(y\right)}\right)}{\sqrt{V_{n+m}^{\left(x\right)}}\sqrt{V_{n+m}^{\left(y\right)}}}\\ =\frac{n-1}{n+m-1}r_{n}^{\left(xy\right)}+\frac{m-1}{n+m-1}r_{m}^{\left(xy\right)}\\ +\frac{1}{n+m-1}\frac{nm}{n+m}\frac{\left(M_{n}^{\left(x\right)}-M_{m}^{\left(x\right)}\right)\left(M_{n}^{\left(y\right)}-M_{m}^{\left(y\right)}\right)}{\sqrt{V_{n+m}^{\left(x\right)}}\sqrt{V_{n+m}^{\left(y\right)}}}. \end{array}$$ (16)

Eq. 16 shows that the correlation between *x* and *y* taken over the full data set is a weighted sum of the correlations $r_{n}^{\left(xy\right)}$ and $r_{m}^{\left(xy\right)}$ taken over the two subsets plus and additional term

$$\Delta r_{n+m}^{\left(xy\right)}=\frac{1}{n+m-1}\frac{nm}{n+m}\frac{\left(M_{n}^{\left(x\right)}-M_{m}^{\left(x\right)}\right)\left(M_{n}^{(y)}-M_{m}^{(y)}\right)}{\sqrt{V_{n+m}^{\left(x\right)}}\sqrt{V_{n+m}^{(y)}}}.$$ (17)

The term $\Delta r_{n+m}^{\left(xy\right)}$ does not depends on the correlation between *x* and *y*, but only on the difference between the mean value of *x* and *y* in the two sub sets. As a consequence, even if $r_{n}^{\left(xy\right)}$ and $r_{m}^{\left(xy\right)}$ are zero, the correlation taken over the merged data set is different from zero if *x* and *y* have not the same average $M_{n}^{\left(x\right)}$ and $M_{m}^{\left(x\right)}$ and $M_{n}^{\left(y\right)}$ and $M_{m}^{\left(y\right)}$ in the two sub sets. This is exactly what happens in the examples shown in Figures 2, 3. Working out the calculations for data in Figure 2 we have:

$$\begin{array}{l} n=m=100 \end{array}$$ (18)

$$\begin{array}{l} M_{n}^{\left(x\right)}=43.2\;M_{n}^{\left(y\right)}=43.2\;M_{m}^{\left(x\right)}=39.9\;M_{m}^{\left(y\right)}=40.1\\ V_{n}^{\left(x\right)}=0.92\;S_{n}^{\left(y\right)}=0.86\;V_{m}^{\left(x\right)}=0.77\;V_{m}^{\left(y\right)}=0.96\\ M_{n+m}^{\left(x\right)}=41.5\;M_{n+m}^{\left(x\right)}=41.6\\ V_{n+m}^{\left(x\right)}=3.5\;S_{n+m}^{\left(x\right)}=3.3\\ r_{n}^{\left(xy\right)}=0.08\;r_{m}^{\left(xy\right)}=0.01\\ r_{n+m}^{\left(xy\right)}=0.76. \end{array}$$ (19)

**FIGURE 4:**
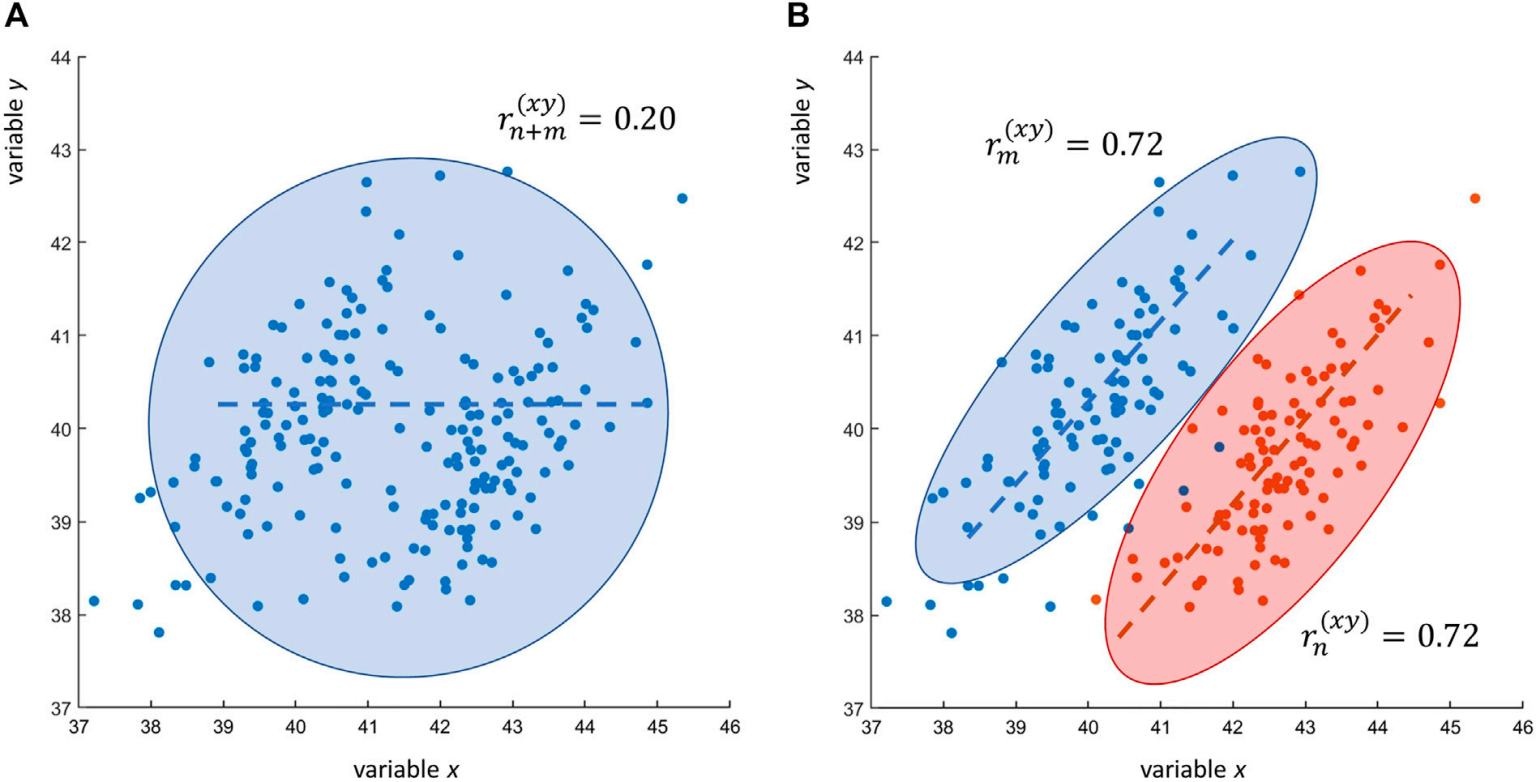
Research scenarios RS1 and RS2. **(A)** Scatter plot of *n* + *m* = 200 observations of two variables *x* and *y*: the Pearson’s sample correlation between *x* and *y* is $r_{n+m}^{\left(xy\right)}=0.20$ . **(B)** The same scatter plot as in Panel A but with data points color coded to highlight the actual data structure: when taken separately the *n* = *m* = 100 observations of *x* and *y* (data set A, blue; data set B: red) are correlated: $r_{n}^{\left(xy\right)}=0.72$ (data set A) and $r_{n}^{\left(xy\right)}=0.72$ (data set B). The low observed correlation $r_{n+m}^{\left(xy\right)}=0.20$ is an artifact due to the merging of two data set containing variables coming from different populations: in this case *x* and *y* come from two independent normal distribution with population means *μ*_*A*_ = (43, 40) and *μ*_*B*_ = (40, 40) and unit variance.

These numerical results are consistent with those we obtained for the synthetic data sets, where uncorrelated observations of *x* and *y* were generated according to:

$$\begin{array}{l} \left(x_{1},x_{2},\ldots x_{n}\right)∼N\left(40,1\right)\;\;\left(y_{1},y_{2},\ldots y_{n}\right)∼N\left(40,1\right)\\ \left(x_{n+1},x_{n+2},\ldots x_{n+m}\right)∼N\left(43,1\right)\;\;\left(y_{n+1},y_{n+2},\ldots y_{n+m}\right)∼N\left(43,1\right), \end{array}$$ (20)

with *n* = *m* = 100. Eq. 16 shows that the contrary is also possible: two variables can be correlated in two different data sets but uncorrelated when the correlation is taken over the two merged data sets: this is shown in Figure 4.

Eq. 16 also explains why the Pearson’s sample correlation is so sensitive to outliers, to the point that one single outlier is sufficient to pull a zero correlation to 1. Having one outlier is the equivalent of having one additional data set (or condition/class/group) with just *m* = 1 observations. As a result, Eq. 16 simplifies to

$$r_{n+1}^{\left(xy\right)}=\frac{n-1}{n}r_{n}^{\left(xy\right)}+\frac{1}{n+1}\frac{\left(M_{n}^{\left(x\right)}-x_{n+1}\right)\left(M_{n}^{\left(y\right)}-y_{n+1}\right)}{\sqrt{V_{n+1}^{\left(x\right)}}\sqrt{V_{n+1}^{\left(y\right)}}}.$$ (21)

**FIGURE 5:**
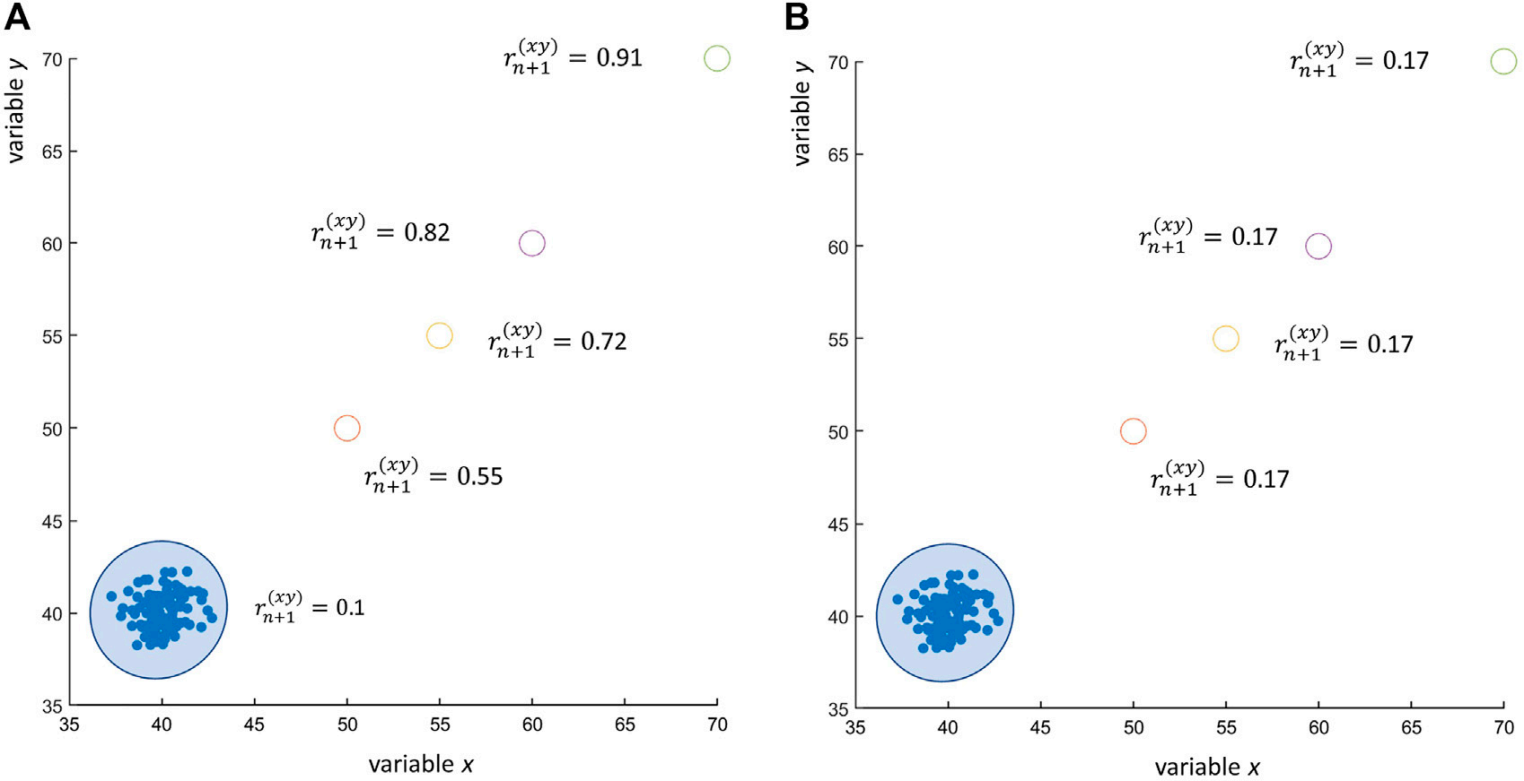
Effect of outliers on the correlation coefficient: 100 observations of two uncorrelated variables *x* and *y* ∼ *N* (40, 1) (observed Pearson’s correlation $r_{n}^{\left(xy\right)}=0.1$ ) to which an outlier observation *x*_*n*+1_, *y*_*n*+1_, with increasing distance from the sample mean (open dots ○), is added. **(A)** The Pearson’s correlation $r_{n+1}^{\left(xy\right)}$ calculated on the *n* + 1 observations (including the outlier *x*_*n*+1_, *y*_*n*+1_) increases with the distance of the outlier *x*_*n*+1_, *y*_*n*+1_ from the sample mean, consistently with Eq. 21. **(B)** The Spearman’s correlation coefficient is not sensitive to the distance of the outlier from the sample mean. However, this is not true if *m* ≥ 2 outliers are present, as shown in Figure 6.

**FIGURE 6:**
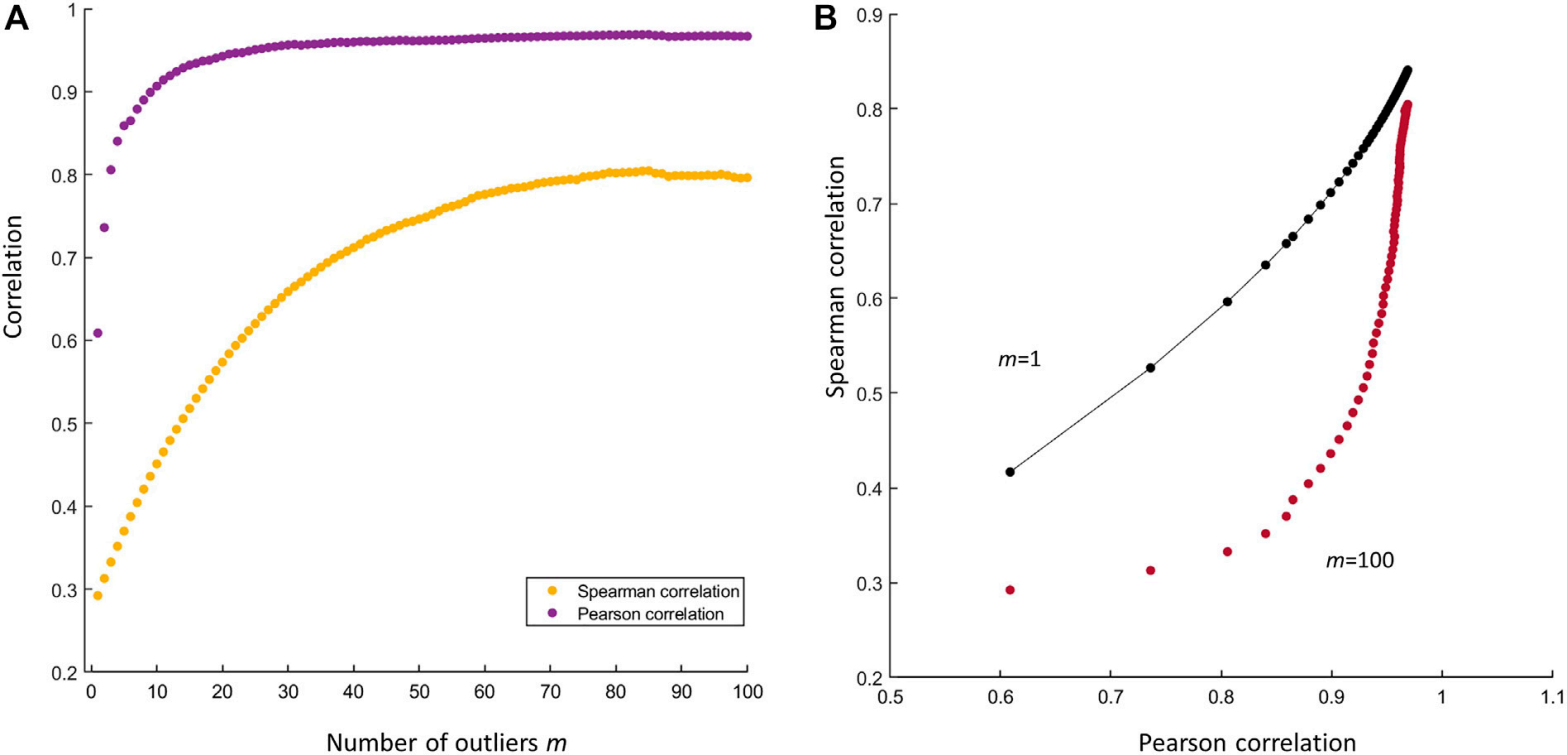
**(A)** Effect of the number of outliers on the Pearson’s and Spearman’s correlation. Both indexes increase with the number *m* of outliers **(B)** Scatter plot of the Spearman’s index against the Pearson’s in presence of *m* = 1 outlier (black dots) and in presence of *m* = 100 outliers (purple dots). The difference between the two indexes is larger when *m* = 1 since the Spearman’s correlation is robust to the presence of that outlier. If the number of the outlier is large (*m* = 100) the difference between the two indexes becomes increasingly smaller since the Spearman index is also affected by the outliers: both indexes record an inflated correlation.

If the outlying observation (*x*_*n*+1_, *y*_*n*+1_) is very distant from the average of the other *n* observations, the resulting correlation can be severely inflated, as shown in Figure 5A. The reader could argue that the use of Spearman’s rank correlation (Spearman, 1904) would have avoided this problem, since Spearman’s correlation is less sensitive to outliers. This is certainly true, as shown in Figure 5B: the presence of an outlier, even if really far from the average of all other *n*, not outlying, observations, does not affect the correlation. However, this argument holds true only in case of a few outliers: if the number *m* of outliers increases, also the Spearman’s correlation increases, albeit less dramatically than in the case of Pearson’s correlation, but still to a significant extent, leading to claim the existence of a correlation between *x* and *y* when *x* and *y* are not correlated at all. This is shown in Figures 6A, B. The agreement between Pearson’s and Spearman’s indexes increases when the number of outliers increase since the Spearman’s index is not robust against a large number of outliers. As shown in the plot, when there are one to two outliers, the Pearson’s coefficient gives a large correlation while the Spearman’s not; and as the number of outliers increase, both agree on a large correlation.

### 2.3 Violation of independence of observations

**FIGURE 7:**
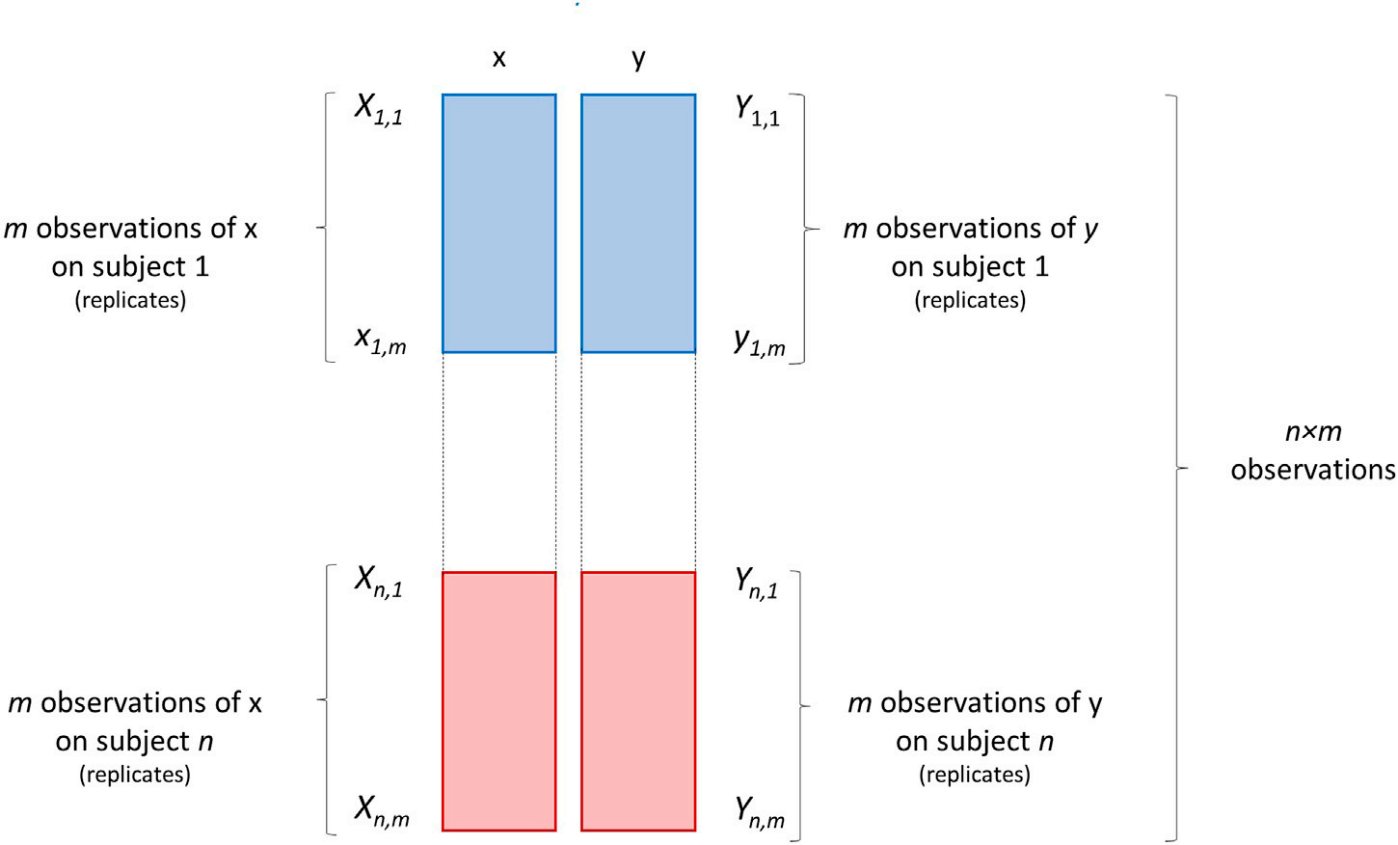
Graphical illustration of a data set containing *m* repeated (or replicated) measurements/observations of two variables *x* and *y* on *n* subjects. Each block of repeated measurements is depicted as a block of different color.

The third scenario RS3 pertains the violation of the assumption that the observations (i.e., the samples on which variables *x* and *y* are measured), are independent, i.e., independence of errors. Working with repeated measures is the most striking case of non-independent observations, since the same subject is measured more times. This scenario is graphically illustrated in Figure 7.

**FIGURE 8:**
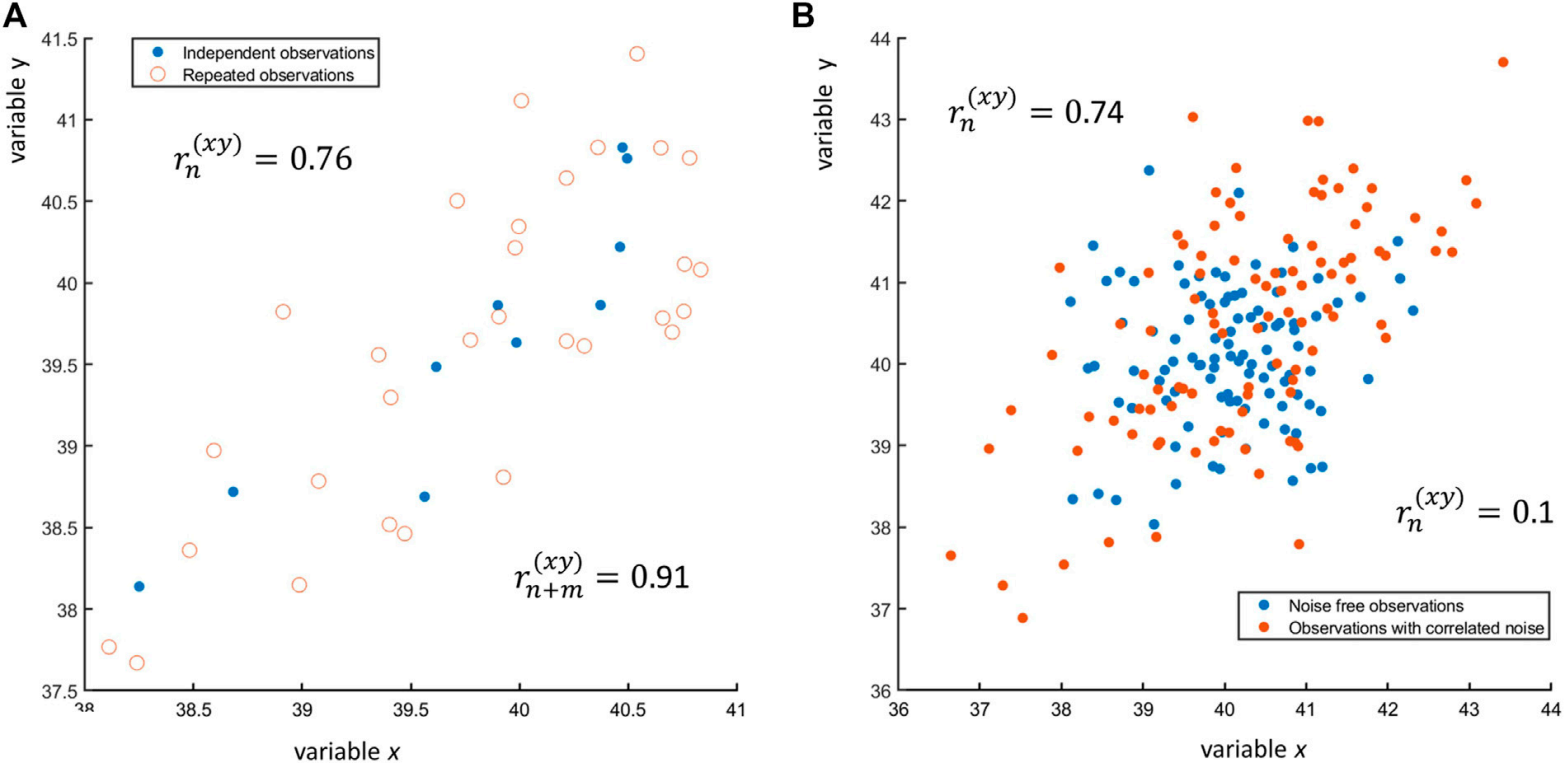
**(A)** Scatter plot of *n* = 10 observations of two variables *x* and *y* (blue dots •) and of three replicates for each observation (red open circles ○). The sample correlation between the *n* = 10 observations is $r_{n}^{\left(xy\right)}=0.91$ . **(B)** if the replicates are considered using a total of n + *m* = 30 observations, the sample correlation reduces to $r_{n+m}^{\left(xy\right)}=0.76$ .

What are the consequences of the violation of the independence of the samples? This depends on the type of dependence present between the observations. Take for instance the case when technical replicates are used (wrongly!) to increase the sample size. In this case more observations (measurements) are available of *x* and *y* on the same sample(s), with the model (a similar equation holds for *y*)

$$x_{i,j}=\mu_{x}+\epsilon_{j},$$ (22)

where *x*_*i*,*j*_ is the *j*th replicate of observation *i*th of *x* and *ϵ*_*j*_ is the replication error. An example of *n* = 25 observations of *x* and *y*, each with three replicates, is shown in Figure 8A: if only one observation per subject is taken (*n* = 25), the correlation between *x* and *y* is *r* ^*(xy)*^ = 0.91, while if all *n* + *m* = 25 + 2 × 25 = 25 × 3 = 75 observations are taken, the correlation is *r* ^*(xy)*^ = 0.76. In general, using replicates considering them as independent observation will lower the value of the correlation coefficient. Repeated measures must be handled carefully with special approaches: there is ample literature on this topic, see for instance (Bakdash and Marusich, 2017) and reference therein.

Dependence of observations can also arise because of reasons that are out of the control of the experimenter, like in presence of correlated measurement noise, where data can be modeled as

$$x_{i}=\mu_{x}+\epsilon_{x_{i}}+\phi_{x}$$ (23)

$$y_{i}=\mu_{y}+\epsilon_{y_{i}}+\phi_{y},$$ (24)

where *ϕ*_*x*_ and *ϕ*_*y*_ are correlated error terms normally distributed with zero mean and given error variance-covariance. The presence of correlated error can induce correlation between two variables that are originally uncorrelated, as shown in Figure 8B. The effect of correlated and uncorrelated error on the Pearson correlation coefficient is discussed in (Saccenti et al., 2020).

Another commonly seen error is the estimation of the correlation using time series data to increase the sample size, which is another violation of the assumption of independent observations. This type of data also needs to be handled carefully, since it is very easy to obtain misleading results: a classic reference on this topic is (Yule, 1926).

## 3 Plot the data rather than blindly trusting correlation values

**FIGURE 9:**
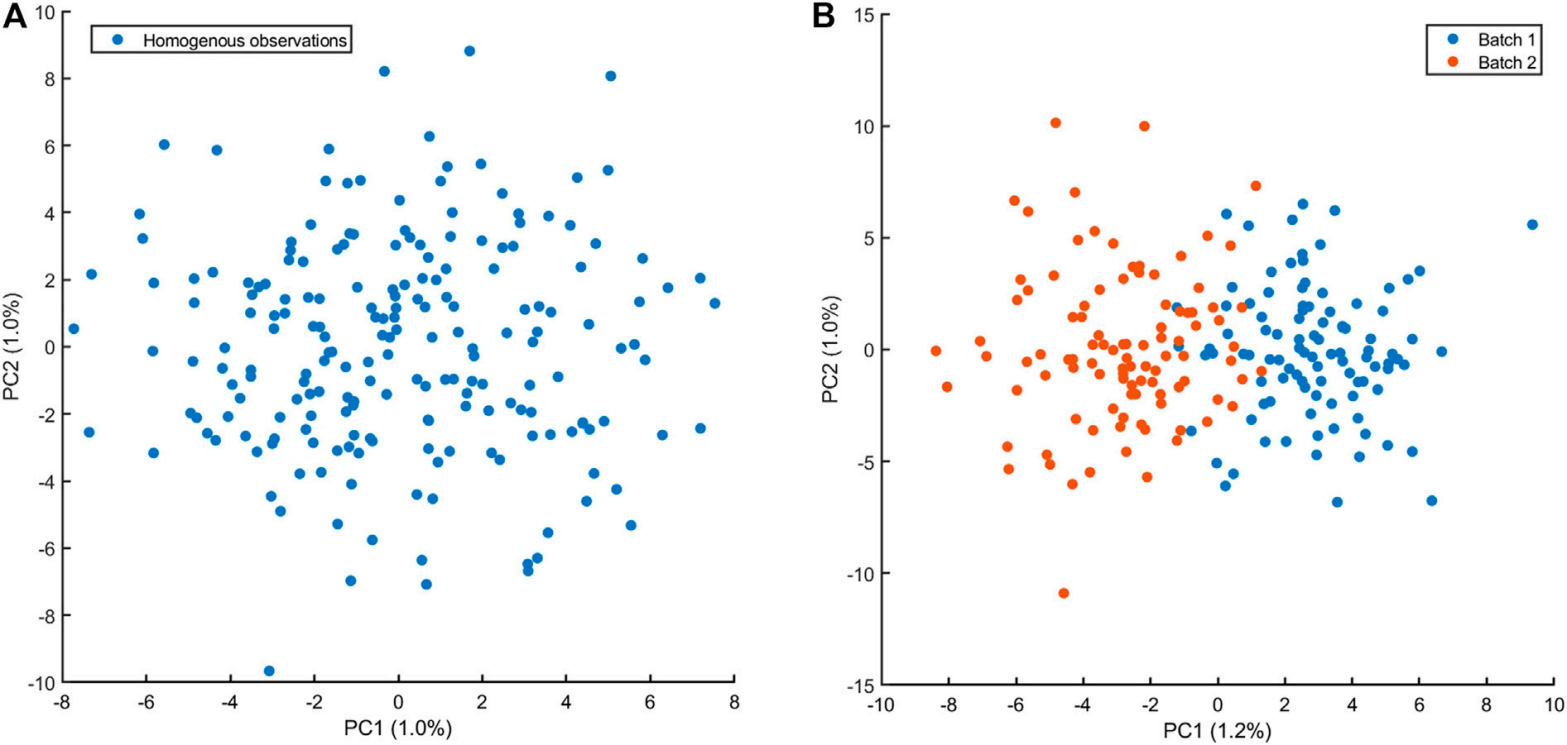
Principal component analysis score plot of *n* = 200 observations of *p* = 1,000 variables. **(A)** Observations are sampled from the same normal multivariate distributions: variables are uncorrelated with unit variance and mean 40. There is no structure in the data and no outliers are visible. **(B)** Observations sampled in presence of batch effect affecting 500 variables which are sampled from a normal distribution with mean 40.2. The batch effect is clearly visible in the PCA score plot, suggesting a violation the assumption from one distribution. Caution must be taken when correlations are calculated!

The examples discussed in the previous section should (hopefully) suggest that plotting the data is a critical step for the analysis, understanding and interpretation of correlations: visual exploration of scatter plots like those shown in Figures 2–4, can easily reveal the presence of outliers and data structures that can point to violation of the assumption of sampling from one population or independence of observations (although the latter can be tricky to spot). In the case of multivariate data when *p* ≫ 2 variables are measured, plotting and visual exploration of all possible correlation plots is usually not a feasible approach, since the number of plots increases as $\frac{1}{2}p\left(p-1\right)$ . In this case, Principal Component Analysis (PCA) (Pearson, 1901; Hotelling, 1933; Jolliffe, 2002) is an extremely valuable tool since it can be used to reduce the dimensionality of high-dimensional data and can highlight the presence of outliers and of (unwanted) data structure that hampers the calculation of the correlation coefficient. An example is given in Figure 9, where the PCA plot of a simulated data set without and with data structure (in this case a batch effect) is shown.

## 4 Conclusion

In this technical note, I have shown some of the consequences of neglecting the assumptions of sampling from one population and independence of observations when calculating the Pearson’s correlation coefficient. I illustrated cases of the violation of these assumptions that originate when data sets coming from different experiments or pertaining different experimental conditions or in presence of batch effects are merged before the calculation of the correlation coefficient. It is shown that this way of proceeding will result in inflation or deflation of correlations: inflation or deflation of correlations. In both cases wrong inference will be made, leading to believe that a correlation exists when it does not, or that a correlation does not exist when it actually does. Similar problems arise when correlations are taken over repeated measures or time series.

The hope is that the reader, after having read and meditated the examples, will be able to recognize those situations where the calculation of the sample correlation is not allowed because of the violations of fundamental statistical assumptions.

## Data Availability

The original contributions presented in the study are included in the article, further inquiries can be directed to the corresponding author.

## Footnotes

1 The notation V ^(x)^ with the use of superscript (x) may seem, at fist, unpractical. Its utility will become evident in the remaining of the paper when more and different quantities are introduced.
